# Cardiovascular Risk Prediction Parameters for Better Management in Rheumatic Diseases

**DOI:** 10.3390/healthcare10020312

**Published:** 2022-02-07

**Authors:** Abhinav Sharma, Ruxandra Christodorescu, Ahmad Agbariah, Daniel Duda-Seiman, Diala Dahdal, Dana Man, Nilima Rajpal Kundnani, Octavian Marius Cretu, Simona Dragan

**Affiliations:** 1 Department Cardiology, “Victor Babes” University of Medicine and Pharmacy, 300041 Timisoara, Romania; sharma.abhinav@umft.ro (A.S.); dannymduda@yahoo.com (D.D.-S.); danaemilia@yahoo.com (D.M.); simona.dragan@umft.ro (S.D.); 2 Department of Occupational Medicine, Municipal Emergency University Hospital, 310030 Arad, Romania; 3 Department 5 Internal Medicine, “Victor Babes” University of Medicine and Pharmacy, 300041 Timisoara, Romania; ruxandra_christodorescu@yahoo.com; 4 Research Center of the Timisoara Institute of Cardiovascular Diseases, “Victor Babes” University of Medicine and Pharmacy, 300041 Timisoara, Romania; 5 General Medicine, “Victor Babes” University of Medicine and Pharmacy, 300041 Timisoara, Romania; ahmad.abo.wael@hotmail.com (A.A.); diala.dahdal@student.umft.ro (D.D.); 6 Department of Functional Sciences, Physiology, Center of Immuno-Physiology and Biotechnologies (CIFBIOTEH), “Victor Babes” University of Medicine and Pharmacy, 300041 Timisoara, Romania; 7 Department of Surgery—I, “Victor Babes” University of Medicine and Pharmacy, 300041 Timisoara, Romania; tavicretu@yahoo.com

**Keywords:** rheumatic diseases, risk stratification

## Abstract

The early detection of cardiovascular disease (CVD) serves as a key element in preventive cardiology. The risk of developing CVD in patients with rheumatic disease is higher than that of the general population. Thus, the objective of this narrative review was to assess and describe updated risk-prediction parameters for CVD in patients suffering from rheumatic diseases, and, additionally, to evaluate therapeutic and risk management possibilities. The processes of recognizing CVD risk factors in rheumatic diseases, establishing diagnoses, and discovering CV risk assessments are currently displeasing in clinical practice; they have a limited clinical impact. A large number of references were found while screening PUBMED, Scopus, and Google scholar databases; the 47 most relevant references were utilized to build up this study. The selection was limited to English language full text articles, RCTs, and reviews published between 2011 and 2021. Multiple imaging techniques, such as ECG, ultrasound, and cIMT, as well as biomarkers like osteoprotegerin cytokine receptor and angiopoietin-2, can be beneficial in both CV risk prediction and in early subclinical diagnosis. Physical exercise is an essential non-pharmacological intervention that can maintain the health of the cardiovascular system and, additionally, influence the underlying disease. Lipid-lowering drugs (methotrexate from the non-biologic DMARDs family as well as biologic DMARDs such as anti-TNF) were all associated with a lower CV risk; however, anti-TNF medication can decrease cardiac compliance and promote heart failure in patients with previously diagnosed chronic HF. Although they achieved success rates in reducing inflammation, glucocorticoids, NSAIDs, and COX-2 inhibitors were correlated with an increased risk of CVD. When taking all of the aforementioned points into consideration, there appears to be a dire need to establish and implement CVD risk stratification models in rheumatic patients.

## 1. Introduction

Autoimmune-inflammatory rheumatic diseases (ARDs) are a group of systemic immune-mediated disorders with the potential to target various joints, bones, and connective tissues [1]. They are also correlated with a higher risk of developing cardiovascular diseases (CVD) [2]. Additionally, the diagnosis of cardiovascular (CV) involvement is challenging due to the widely varying clinical presentations of CVD, as symptoms range from mild to life threatening [3]. Furthermore, early detection is crucial, as it helps minimize the resources required in treatment, which lowers the financial burden on the healthcare system [4]. Thus, evaluating the prevalence of CV involvement is of great clinical value as a first step towards individual risk delamination and stratification. In patients with systemic rheumatic diseases, the CVD risk is not solely conditioned by the prevalence of traditional CV risk factors, which include age, sex, smoking, family history, dyslipidemia, obesity, hypertension, and diabetes mellitus [5,6], but, also, by an increased genetic risk, long-term uptake of medications, and chronic inflammation [7]. 

Autoimmune-inflammatory rheumatic disorders, particularly ankylosing spondylitis (AS), systemic sclerosis (SSc), systemic lupus erythematosus (SLE), and rheumatoid arthritis (RA), have been associated with early accelerated atherosclerosis (ATS) [8]. Cardiac autonomic neuropathy, arrhythmias, microvascular dysfunction, and non-ischemic heart failure are also emerging as major contributors to the broad CV involvement; especially considering that inflammatory cells have the ability to directly impact the entire CV system [5,9]. Of further note, some of the medications used in treating rheumatic diseases can have adverse effects on the CV system, whereas others might prove to be beneficial [7]. 

Quality of life (QOL) is another factor that can play a pivotal role in any disease, which makes it essential to assess the patient’s mental and physical health. In cases of CV diseases such as atrial fibrillation, irregular rhythm causes poor QOL [10,11]. Similarly, inflammatory rheumatic diseases, in which chronic pain, decreased joint mobility, and limitations in routine activities occur constantly, can excessively affect the QOL [12]. Improving QOL can help improve the overall disease outcome, which causes patients to be more compliant to treatment. 

## 2. Aims

We used the Narrative Review methodology to classify and assess the latest findings in the literature pertaining to Cardiovascular (CV) manifestations in various rheumatic diseases, in order to shed light on each manifestation individually while stratifying and analyzing CV risk prediction models, therapies, and risk management practices pertaining to CV involvement in rheumatic diseases.

Search strategy: Thorough searches were conducted in PUBMED, Scopus, and Google scholar using the following keywords: rheumatic diseases, cardiovascular involvement, ATS, autoimmune disease, CV risk stratification, and disease prevention in rheumatic diseases. The resulting articles were limited to English language full text articles, RCTs, and reviews which were published between 2011 and 2021.

Article eligibility and data extraction: The assessment of these studies was done independently by two blinded reviewers. Any disagreements between them were resolved by consensus using predefined eligibility criteria. All of the articles found in the search were reviewed using their titles and abstracts; the articles which were not relevant were excluded. 

We applied two levels of screening. On the first level, we reviewed titles and abstracts to exclude irrelevant studies; on level two, we reviewed full-text articles to determine the relevance of the studies.

We included a study (1) if the abstract was available, (2) if it contained original data, (3) if it used accepted classification criteria for each rheumatic disease, (4) if it discussed all CV risk factors (traditional and/or nontraditional), and/or (5) if it examined cardiovascular prediction models or preventive strategies, and/or (6) if it analyzed management strategies and examined the effects of the drugs available for the treatment and management of CV manifestations.

We excluded: case reports, topics not related to cardiovascular involvement in rheumatic diseases, articles with insufficient data or articles showing results with lack of statistical significance, and articles that did not meet the inclusion criteria.

We used the ENDNOTE program to archive our search and create the list of abbreviations. 

## 3. Results

**Figure 1:**
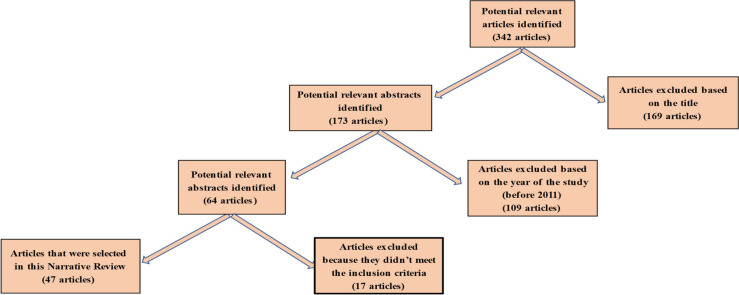
Flow diagram of the search process.

The search of electronic databases resulted in 342 potential articles. Of these, 169 were excluded based on their titles. From the remaining 179 articles, 109 were excluded based on the year of study (before 2011), and 17 more articles were excluded because they did not meet the inclusion criteria. The remaining 47 articles were selected for this review. The selection process is represented by the flow diagram in Figure 1.

## 4. Discussion

The pathophysiological association between RA and CV risk is linked to the traditional risk factors and vascular damage, both of which trigger inflammation in a vicious cycle. In addition to traditional cardiovascular risk factors, which include age, gender, family history, smoking, sedentary lifestyle, and dyslipidemia, genetic risk has also been shown to play a role when defining global cardiovascular risk. 

Chronic inflammation combined with autoimmunity may lead to accelerated atherosclerosis. Disease severity scores and markers of high disease activity are also linked to increased cardiovascular risk [13].

This process is triggered by inflammatory cells, autoantibodies, adhesion receptors, chemokines, cytokines, and proteases, which are involved in cascades directly affecting all structures of the cardiovascular system, from the myocardium to the cardiac valves, conduction system, and vasculature [14]. Additionally, medications used to treat RA, like DMARDs leflunomide and cyclosporine, glucocorticoids, NSAIDs, and cyclo-oxygenase II inhibitors, may be involved in the development of hypertension in these patients [15].

When predicting cardiovascular risk in ARDs, although it is a well-known fact that ARDs patients hold high CV risk, numerous patients continue to receive inappropriate CV prevention and care. This is most likely due to a combination of reasons; many rheumatologists have prioritized the prevention of musculoskeletal symptoms or other ARD-specific problems while, resultingly, overlooking the CV prophylaxis. Furthermore, there is an unawareness regarding CV risk in ARDs patients and in the general population, and, consequently, less patients present at the cardiology clinics for routine follow-ups if they are diagnosed with rheumatic illnesses. Moreover, the diagnosis of CVD in the setting of ARDs is often difficult, due to various factors, including: the lack of symptoms (asymptomatic patients) or atypical symptoms; the physician’s misinterpretation of chest pain as emanating from the musculoskeletal system rather than the CV system, due to the presence of a high inflammatory state; and a lack of suspicion of CVD in patients who do not present traditional risk factors (TRFs), especially in the young and/or female patients. In addition to the absence of clinical evidence, there are a lack of proper guidelines for CV prophylaxis in ARDs patients, and this further minimizes the possibility of enrolling the patients in cardiovascular pathology prevention programs. This lack of proper guidelines also diminishes the possibility of making the regular follow-up schedules mandatory, which would help detect, at an early phase, the CV involvement in ARDs patients [16].

### 4.1. Risk-Prediction Models

Assessment of CV risk is well-established in RA patients, and to a slightly lesser extent in other ARDs patients, suggesting that CV risk evaluation should be a part of routine practice with all patients. Clinicians should be able to recognize the patients who are most at risk and adjust their treatment accordingly. Unfortunately, the CV risk in RA patients is still undervalued in clinical practice [17]. Despite improvements in risk stratification and guidelines from the European League Against Rheumatism (EULAR) Task Force [18], CV risk assessment remains unsatisfactory [7]. Several algorithms, such as the QRISK^®^2 (EMIS and the University of Nottingham, UK), the Reynolds Risk Score, Framingham Risk Score, and SCORE chart, have been developed in the last 12 years to help estimate the risk for CVD. These risk models are primarily based on randomized cohort studies conducted in the general population, posing the question of whether they should be included in ARDs patients or not. QRISK^®^2 was built in a community that included RA patients; the algorithm includes RA as an additional independent risk factor. CRP values are included in the Reynolds Risk Ranking, although not in the range found in high-grade inflammatory diseases [7]. None of the previously mentioned risk assessment tools provide information pertaining to the impact of inflammation and anti-rheumatic drugs on lipids and other TRFs, nor do they take into account systemic variations between ARDs populations, comprising mainly of females from certain age groups, compared to the general population. Many efforts have been made to resolve these issues; for example, the EULAR Task Force has suggested that, when measuring CV risk in RA patients, risk models should be adjusted by adding a multiplication factor of 1.5 when a patient fits two or more of the following three criteria: (a) positive for anti-citrullinated protein antibodies (ACPA) or rheumatoid factor, (b) duration of the disease is over 10 years, and (c) occurrence of extra-articular manifestations [19].

The QRISK^®^2, as well as 2012 ESC guidelines, consider RA as an independent CV risk factor in their models; nonetheless, the existence of RA has no impact on clinical management in the ESC protocols in comparison to QRISK^®^2 [20]. However, none of these methods can be considered competent in increasing the precision of assessments of CV risk in ARDs patients when compared to models that do not incorporate RA, and, therefore, risk-stratification models for this population of patients need to be improved extensively [21,22]. The novel Pooled Cohort Equations were used to measure the 10-year probability of atherosclerotic CV disease in the American Heart Association (AHA) and in the 2013 recommendations of the American College of Cardiology (ACC) [23]. When compared to prior recommendations, this estimate significantly improved the number of RA patients that will be eligible for cholesterol-lowering statin therapy, but it did not enhance the estimation of CV risk [24,25]. Imaging tests and special biomarkers can facilitate early detection. Alternatively, ARDs-based risk-prediction models would not be required if all patients with ARDs were subjected to primary prevention by maintaining specific lipid and blood-pressure threshold levels, as seen in the case of diabetes, where specific protocols are strictly followed to improve long term prognosis [20]. Similar strategies would be easier to implement than the process of designing specific models and evaluating their reliability, efficacy, and cost-effectiveness in different communities [7].

### 4.2. Imaging Techniques

The increasing rate of asymptomatic ATS in RA patients can mislead physicians while evaluating CV risk in this group. Many techniques, including Echocardiography (ECHO), carotid ultrasonography, cardiac computed tomography (CT), cardiac magnetic resonance imaging (MRI), and positron emission tomography (PET-SCAN), are defined as diagnostic instruments capable of detecting CV complications in inflammatory rheumatic disease patients, and they often lead to a better prognosis. Simultaneously, their findings can provide additional risk stratification values for asymptomatic patients in the context of primary prevention. Several studies have demonstrated the effectiveness of novel echocardiography techniques (Tissue Doppler Imaging), especially global longitudinal strain through speckle monitoring, in assessing subclinical cardiac involvement and left ventricular diastolic dysfunction [5]. ECHO is a primary tool for detecting CV involvement, and it also assists in monitoring the therapeutic effects and disease progression [1]. A noninvasive method for detecting ATS plaques in the carotid artery is carotid ultrasound investigation [7]. The existence of carotid ATS can be used to predict the presence of ATS in the coronary arteries. Furthermore, with the help of carotid ultrasonography, we can obtain the carotid intima-media thickness (cIMT) measurements. When compared to non-RA individuals, cIMT is elevated in those who have RA, and this elevation is associated with a higher rate of CV events [20]. The existence of carotid plaques (CP) was identified in the 2012 ESC guidelines as equivalent to having CVD, and this implies that the use of ultrasonography for risk evaluation would result in a more accurate selection of patients at high risk than the current existing models which do not use imaging. Increased cIMT was defined as ≥0.9 mm. CP was defined as a focal narrowing ≥0.5 mm of the surrounding lumen or a cIMT ≥ 1.2 mm. 

Wah-Suarez et al. found that carotid plaque was over two times more present in RA than in controls [26], and Rueda-Gotor et al. confirmed that the carotid ultrasound was more sensitive in detecting high cardiovascular risk axial spondylitis than the coronary artery calcification score [27].

Similarly, in RA patients, adding carotid ultrasonography resulted in the increasing sensitivity of detecting a high CV risk population when compared to the adjusted EULAR SCORE alone [28]. CV risk was calculated according to the modified EULAR systematic coronary risk mSCORE chart for RA by application of a multiplier of 1.5 in patients fulfilling ≥2 of 3 specific criteria. Ultrasonographic evaluation of patients with psoriatic arthritis who were stratified by Framingham Risk Score resulted in the reclassification of a significant number of patients into higher CV risk groups [29]. In a report pertaining to the use of carotid ultrasonography in RA patients, this strategy led to the classification of 39% of patients as having a high risk of CVD, while the Framingham Risk Score estimated merely 7% to have a high risk [30]. 

Combining the QRISK3 and the EULAR modified systematic coronary risk evaluation (mSCORE) algorithms may further optimize the identification of people with rheumatoid arthritis (RA) at high risk for carotid plaques [31].

While these findings indicate that using carotid ultrasonography in patients with ARDs could strengthen CV assessment, it is yet to be proven that this incorporation would result in reduced CV risk in these patients [32]. Carotid ultrasonography has its drawbacks, such as its reliability and accessibility; therefore, several matters should be considered before this imaging technique is recommended [7].

### 4.3. Biomarkers

In parallel to the imaging methods, the value of a variety of biomarkers was examined in the CV risk prediction. These biomarkers include inflammation markers, genetic factors, endothelial function, and immunological markers. In patients with RA, for instance, levels of osteoprotegerin cytokine receptor, also known as osteoclastogenesis inhibitory factor or tumor necrosis factor receptor superfamily member 11B, are correlated with the presence of CVD [33], as these have a correlation with carotid ATS and endothelial activation [34]. Angiopoietin-2 is another endothelial function marker that is correlated with CVD in RA patients [35]. Patients with RA also present with high levels of pro-inflammatory cytokines such as interleukin (IL) 1, IL-6, and tumor necrosis factor α (TNF-α). These pro-inflammatory cytokines trigger systemic inflammatory responses, and they inhibit endothelial NO synthesis, leading to arterial stiffness, which adds to CV risk.

The efficacy of B-type natriuretic peptide (heart failure marker NT-proBNP) as a predictor for CV risk in the case of rheumatic disease has also been evaluated [36]. Some recent studies have been undertaken on the association between NT-proBNP and inflammation in rheumatic diseases, as up-regulation of neurohormonal axis is linked with inflammation. These patients are considered to be at high risk for developing pulmonary hypertension, because, in connective tissue, disease high right ventricular overload determines increased NT-proBNP synthesis [36]. Serum uric acid levels have been linked to hypertension, renal failure, and CVD in RA patients, although it is uncertain if these correlations are related to unique pathogenic pathways or are an epiphenomenon [37]. In RA patients, mean platelet volume and microalbuminuria are correlated with hypertension, but the usefulness of these measures in predicting CV risk is unclear [38]. Symmetric and asymmetric dimethylarginine are possible biomarkers of inflammatory vascular injury and CVD In RA [39,40]. The use of biomarkers in risk-assessment tools in order to enhance CV risk stratification was shown in a large European population, where enhanced measurement of the combination of N-terminal pro-brain natriuretic peptide, troponin I, and CRP, resulted in an improved 10-year risk assessment when compared to TRF model alone. It is unknown how useful these biomarkers are for risk prediction in the presence of ARDs. The effect of ARDs activity and treatment on biomarker levels has yet to be evaluated, and this complicates the determination of their value. ARDs cohorts are much smaller, insufficient to validate biomarkers against particular end points for the entire population, and this implies that international cooperation would be beneficial [7].

#### Cardiovascular Risk Management

The process of risk identification opens up possibilities for disease prevention. Three core concepts are considered by the rheumatologists while managing the CV comorbidities in ARDs patients: nonpharmacological treatment of CV risk factors, pharmacological treatment of CV risk factors, and strict monitoring of disease progression (Figure 1). Unfortunately, the prevalence of CVD in RA patients is undervalued, and, resultingly, prevention measures are provided at a lower rate than in the general population.

### 4.4. Lifestyle Interventions

The first steps in CV risk control should be lifestyle changes, as they are the most important non-pharmacological interventions in CV prevention in ARDs and chronic inflammatory disorders. Improving the QOL should be one of the main goals. Patients should be encouraged to stop smoking, and they should be encouraged for including daily physical exercise in their schedule. Aerobic activity and physical fitness provide significant impacts on the endothelial system, both acutely and chronically [41]. Exercise has multiple CV benefits in ARD patients, according to evidence from lifestyle programs [42]. Regulated exercise therapy improves cardiorespiratory health as well as macrovascular and microvascular functionality, and, indeed, it reduces CV risk. Exercise, in fact, can invert endothelial dysfunction by enhancing anti-oxidative processes and increasing vascular endothelial growth factor, endothelial progenitor cell, endothelial nitric oxide synthase (eNOS), and prostaglandins synthesis, thereby boosting angiogenesis, local blood flow, and endothelial growth [16]. The higher eNOS activity is accompanied by a decrease in the up-regulation of adhesion molecules, monocyte chemoattractant protein-1, and endothelin-1, which have all been shown to favor the infiltration of inflammatory cells, especially T cells and monocytes, to the capillary endothelial wall, thereby facilitating atherosclerotic wall injury. Finally, it has been shown that daily physical exercise has a significant systemic anti-inflammatory effect. Undoubtedly, mild muscular exercise decreases the size of adipose tissue, which can lead to an increase in pro-inflammatory molecules like (CRP) and (IL)-6 [42]. Muscular exercise enhances overall muscle hypertrophy and coordination, decreases adipose tissue, and enhances the immune response in RA patients, especially those with structural joint injury. Furthermore, regular exercise has been shown to decrease disease severity and activity, as it is very beneficial for different disease outcomes [43]. Although the CV benefits of physical activity are well documented, there are a few studies that contradict the conclusions pertaining to associations between exercise and subclinical markers of ATS, or those pertaining to the impact of exercise on CV outcomes in patients with ARDS [16]. In a recent study involving women with SLE, poor physical activity was linked to an increased risk of subclinical ATS, as measured by increased carotid IMT and plaque development. Furthermore, in the same population, less physical activity was correlated with the existence of pro-inflammatory HDL, a molecule recently implicated in the induction of subclinical ATS in SLE. Previous research suggests that physical activity may contribute to a decrease in the inflammation associated with ATS, and to influencing inflammation markers in these patients [16,42]. It should be noted: people with RA and other chronic systemic inflammatory disorders are known to have a lower degree of physical activity due to articular discomfort and joint deformity. Given the proof of the importance of physical exercise in suppressing disease activity and optimizing disease outcomes, routine physical activity should be incorporated into the basic treatment of patients with chronic ARDs. Even so, further research is needed to examine and analyze the effects of physical exercise and muscle fitness on CV outcomes in these patients [44].

The Mediterranean diet or plant-based diets, rich in whole grains, fruits and vegetables, and low in saturated fats and sodium, might help reduce symptoms associated with rheumatoid arthritis. There is a strong scientific rationale for the use of dietary n-3 fatty acid supplementation to modulate inflammation [45]. A recent review revealed a significant reverse association between fish consumption and risk of RA [46].

### 4.5. Pharmacological Interventions

#### 4.5.1. Lipid Lowering Drug Treatment

Chronic ARD patients have an altered pro-atherogenic lipid profile distinguished by low HDL-c levels and elevated LDL-c, total cholesterol (TC), and triglyceride levels. Furthermore, higher levels of oxLDL and lower levels of small dense LDL-c were found in untreated active RA patients, which is a potential CV risk factor associated with an increased risk of ATS [47]. Numerous laboratory trials have conclusively shown that lipid-lowering medications have anti-inflammatory and immunomodulatory effects [16]. Statins are capable of inducing apoptosis in RA synoviocytes, and they inhibit the synthesis of T helper 1 cytokine in inflamed joints, especially IL-2 and interferon-α. After treatment with statins, endothelial cells produce more eNOS and less endothelin, resulting in less endothelial cell activation, which is an early phase in atherogenesis. Furthermore, statins lower the level of circulating CRP and other pro-inflammatory molecules, inhibit inflammatory cytokine production, and have a plaque-stabilizing effect. Recent studies examined the impact of statins in patients with chronic ARDs, especially RA [48]. 

Patients with high blood lipid levels, who were controlled with lipid-lowering drugs, had less of a chance of developing RA than subjects who were not handled with statins, implying that this class of drugs may play a protective role against RA progression in subjects with impaired lipid profiles. Evidence for the beneficial impact of statins on disease progression is rising; this evidence is being supported by the immunomodulatory process. In RA patients, the use of simvastatin and atorvastatin has been shown to change indirect measures of subclinical ATS. Following a brief duration of statin administration, some RA cohorts showed a substantial improvement in systemic arterial stiffness and endothelium-dependent vasodilation, and these are all considered to be indirect indicators of yet reversible endothelial dysfunction [22]. A thorough assessment of the risk-to-benefit ratio of long-term statin treatment should always be taken into account. Furthermore, prior to statin administration, patients’ age and consequent CV risk factors, clinical activity, concurrent medications, comorbidity, and long-term prognosis should be adequately assessed [49].

#### 4.5.2. NSAIDs and Cyclooxygenase-2 Inhibitors

While the advancement of synthetic and biologic DMARDs has resulted in significant reductions in the use of COXIBs and NSAIDs in the treatment of ARDs, these agents continue to play important roles in disease control. Nevertheless, in the general population, the use of COXIBs and NSAIDs is linked to an increased risk of CVD. Following the use of rofecoxib and valdecoxib, a subgroup study classified RA patients as being a CV risk group, and this eventually led to the withdrawal of these drugs from the market. CV risk in ARD patients following treatment with rofecoxib on its own, was observed in a study that was published in 2015. Notably, therapy with NSAIDs and COXIBs may be effective in many RA patients, as it may improve physical activity and reduce inflammation [50].

#### 4.5.3. Glucocorticosteroids

Although glucocorticosteroids have a confusing and controversial association with CV risk, they are one of the most commonly prescribed drugs for rapid management of inflammation. They are, indeed, very successful in reducing inflammation, which is linked to an increased risk of CV disease, but, on the other hand, they can trigger hypertension, raise insulin resistance from baseline values, cause metabolic syndrome, and alter lipid profiles, all of which simultaneously increase CV risk [51]. Higher incidence of arterial stiffness, endothelial dysfunction, plaque formation, and high mortality rates were correlated with RA patients who used high-doses of glucocorticosteroids for a long-term (a dose of >7.5 mg prednisolone equivalent a day), but the net CV impact of glucocorticosteroid exposure remains uncertain [52].

#### 4.5.4. Anti-Rheumatic Therapy

Due to the obviously strong connection between ATS, inflammation, and immune dysregulation, interest has recently shifted to the possible beneficial effects of biologic agents and conventional disease-modifying drugs on various CV risk factors, such as subclinical markers of ATS, lipid profile, and metabolic syndrome. In general, processes such as close monitoring of disease development, as well as early quick suppression of the inflammatory process, are now considered effective in CV disease risk prevention in subjects with ARDs [7].

#### 4.5.5. Non-Biologic DMARDs

Methotrexate (MTX), the key RA treatment, has received the most attention in studies investigating the impact of non-biologic DMARDs on CV risks. Present findings suggest that MTX use is correlated with a lower risk (ranging from 40% to 70%) of CV events and deaths; this is mostly due to a lower risk of acute coronary events and hospitalization caused by HF. MTX therapy appears to decrease CV risk in RA patients in comparison to patients who do not receive MTX, but the mechanisms behind this preventative property remains unknown [50]. In terms of MTX efficacy, drug-induced suppression of systemic inflammation appears to be the most important mechanism for reducing CV morbidity and mortality in these patients. This inflammatory theory is currently being investigated by administering low doses of MTX to patients with chronically high CRP and a previous MI incidence to see whether MTX can play a role in reducing the risk of secondary CVDs [16,53].

#### 4.5.6. Biologic DMARDs

In patients with RA, anti-TNF treatment decreases inflammation and it is linked to reduced CV risk when compared to non-biologic DMARDs. In these patients, anti-TNF treatment shifts lipid levels from baseline, increasing TC, HDL-c, triglycerides, and, probably, LDL cholesterol [50]. These modifications are most likely due to a normalization of lipid levels caused by inflammation suppression. At high doses, these medications can promote HF and decrease cardiac compliance in patients with mild to serious chronic HF [52]. Anti-TNF- agents, on the other hand, tend to improve vascular function, especially endothelial function and aortic stiffness; findings on carotid IMT improvement have been inconsistent. Furthermore, TNF blockade appears to preserve HDL cholesterol’s antiatherogenic effects. Nevertheless, these beneficial effects on vascular function are temporary, reversible, and are found predominantly in anti-rheumatic therapy responders [54]. These findings indicate that, in the long run, prospective longitudinal trials are required to determine the precise role of anti-TNF-blockade in the prevention of ATS. Tocilizumab, a monoclonal antibody against the IL-6 receptor that activates the IL-6 signaling pathway, has also been linked to lipid modifications in clinical trials [16]. A meta-analysis found that, when compared to placebo, treatment with tocilizumab (also with tofacitinib) resulted in higher amounts of TC, HDL-c, and LDL-c in RA patients [7]. Tocilizumab has a stronger impact on lipid levels than other biological drugs, and this is not surprising given that IL-6 impacts serum lipid levels by fatty acid redistribution into peripheral tissues [53]. It is worth noting, however, that anti TNF- therapy seems to be capable of decreasing IR, CRP, and IL-6 while increasing HDL-c. Interestingly, anti-TNF- drugs have been shown to have a selective effect on T-cell subsets which are believed to be involved in plaque development [7]. In ATS plaques that form in unstable angina patients, CD4+ cells without the co-stimulatory receptor CD28 (CD4+ CD28null T cells) are formed and expanded in the peripheral blood of these patients as well as a subset of RA patients. In RA, their expansion is correlated with increased cIMT, suggesting that this may be a marker of subclinical ATS. In this situation, infliximab has been shown to suppress the expansion of these potentially harmful T cells in RA peripheral blood [16].

## 5. Conclusions

Numerous efforts have been made to improve the cardiovascular risk assessment in rheumatic patients: the EULAR guidelines, which recommend a modified SCORE (mSCORE) in RA patients with a duration of disease of more than 10 years; the QRISK2 and QRISK3 algorithms, which use RA as a CVD risk predictor; and various risk prediction models that include markers such as disease activity, duration, and disability index have predicted the risk of composite CVD events such as MI, stroke, and death during the follow-up period of 3 years. All of these calculators have demonstrated controversial results [15,22,55,56].

Cardiovascular involvement in autoimmune rheumatic disease patients often goes undetected in the initial phases of the disease. The majority of the manifestations tend to be clinically silent, while early detection and proper management of these manifestations remain crucial in the control of rheumatic patients, as they can help lower the mortality rates. However, prevention is the key, both for slowing the progression of the disease and, also, for achieving a better QOL, which can be achieved by lifestyle changes, encouraging patients to quit smoking, promoting awareness programs explaining the benefits of physical exercise on the CV system, and by explaining how exercise can slow down the progression and decrease the severity of disease. CV risk evaluation should be a part of routine clinical practice. Rheumatologists should recognize higher risk patients in order to adjust their treatments accordingly. Effective control of traditional CV risk factors is essential, and the use of imaging techniques such as echocardiography, ultrasound, and electrocardiography should be a part of routine checkups. Effective systemic inflammation management, and a thorough comprehension of the complicated autoimmune processes involved in ATS, will likely be needed for the prevention of CV disease in systemic autoimmune diseases. RA therapy appears to be linked to certain vascular functional improvements, but the relationship between these dysfunctions and the activity of the disease still remains unclear. Therefore, future research should focus on the development of effective screening protocols for the early identification of patients at a higher risk, as these can improve long term prognosis by intervening at a faster rate. 

## Data Availability

Not applicable.
